# Therapeutic effect of intradiscal pulsed radiofrequency on internal disc disruption: A case report

**DOI:** 10.1097/MD.0000000000028831

**Published:** 2022-02-11

**Authors:** Dong Hyuck Kim, Kyung Wook Jeong, Wonyeong Jo, So Young Lee, Jung A Im, Jin Yong Jung

**Affiliations:** Department of Anesthesiology and Pain Medicine, School of Medicine Daegu Catholic University, Daegu, Republic of Korea.

**Keywords:** case report, discogenic low back pain, intradiscal stimulation, pulsed radiofrequency

## Abstract

**Rationale:**

Discogenic low back pain often persists despite medication and medical intervention. In this study, intradiscal pulsed radiofrequency (PRF) was performed in a patient with discogenic low back pain who did not respond to oral medication, posterior medial branch block, epidural steroid injection, and percutaneous epidural adhesiolysis.

**Patient concerns:**

A 28-year-old woman visited a pain clinic complaining of low back pain that was scored 8 out of 10 on a numerical rating scale. Her pain was present in any position throughout the day and worsened in the sitting position.

**Diagnoses:**

Magnetic resonance imaging showed L5-S1 internal discal disruption. Based on the medical history, physical examination, and magnetic resonance imaging, we determined that her pain originated from the L5-S1 disc.

**Interventions:**

We performed an intradiscal PRF on the affected disc under C-arm fluoroscopy guidance. PRF was performed at 5 Hz, 20-ms pulse width, and 70 V for 15 minutes while ensuring that the electrode tip temperature was maintained below 42°C.

**Outcomes:**

Immediately after the procedure, the patient's pain subsided. At the 1-month follow-up visit, the patient reported complete relief of her low back pain. The Oswestry disability index, which indicates the degree of disability, improved significantly. She also reported that she could sit for long periods because the pain was reduced. No adverse effects from the procedure were found.

**Lessons:**

Applying intradiscal PRF seems an effective and safe technique for treating discogenic low back pain.

## 1 Introduction

Back pain is one of the most common complaints reported in outpatient clinics, occurring in up to 80% of people during their lifetime. Several structures may cause low back pain, including muscles, ligaments, nerves, joints, and intervertebral discs. Manchikanti et al^[1]^ reported that in 85% of patients without disc herniation and neurological defects, the cause of low back pain could not be diagnosed accurately; however, it is estimated that 39% of low back pain patients have intervertebral disc problems. Discogenic low back pain is typically a non-radiating neuraxial pain due to degenerative changes in the intervertebral disc and is mainly caused by annular fissures.^[2]^ Provocative discography is used as a diagnostic method to confirm the discal pain origin, although its usefulness has been questioned due to side effects such as invasiveness, radiation exposure, risk of infection, and induction of disc regression.^[3]^

Thorough history-taking, physical examination, and imaging studies are essential for diagnosing disc pain. In particular, the high-intensity zone (HIZ), a sign of annular fissure, is important for the clinical diagnosis of discogenic low back pain. HIZ is a small hyperintense lesion on the posterior area of the annulus visible on T2-weighted magnetic resonance imaging (MRI), indicating inflammation, neovascularization, and granulation tissue.^[4]^ HIZ was considered a critical marker of internal annulus disruption; however, since the use of MRI has become common, additional studies have revealed that the presence of HIZ is not essential for the diagnosis of internal disc disruption (IDD).^[5,6]^ Therefore, even when HIZ is absent, discogenic low back pain can be suspected based on clinical symptoms and MRI findings. Hence, pulsed radiofrequency (PRF) is possibly effective in these cases as well.

Intradiscal radiofrequency (IDRF) was first introduced in 1997 to treat discogenic pain.^[7,8]^ IDRF uses a heated electrode that coagulates the nerve fibers in the annulus, seals the annular fissure, and reduces inflammatory molecules in the fissures.^[9]^ Conventional radiofrequency (CRF) applies electrical stimulation and high temperature to the targeted nerves or tissues. In contrast, PRF minimizes thermal damage to tissues by introducing a resting time between stimulation impulses and controls the pain signals with electrical stimulation.^[10]^ Several studies have shown that PRF is effective in modulating discogenic pain.^[11,12]^ Here, we report the effect of intradiscal PRF in a case of suspected IDD based on MRI findings and clinical symptoms, combined with a literature review.

## 2 Case report

### 2.1 Patient information

**Figure 1:**
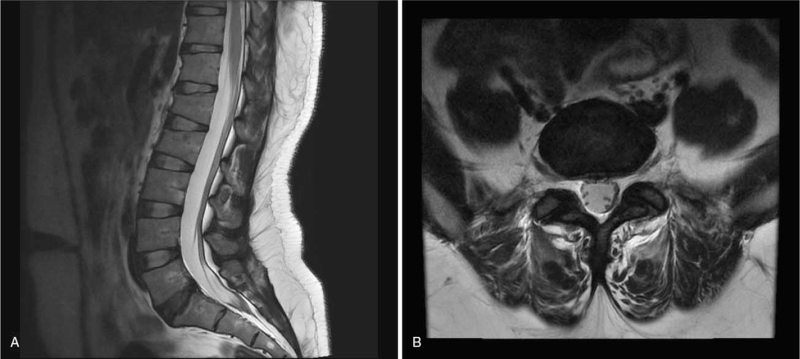
Sagittal and axial T2-weighted lumbar spine magnetic resonance imaging at 2 months after pain onset showing disc bulging and annular fissure without a high-signal intensity zone at L5-S1.

In November 2020, a 28-year-old woman visited the Pain Medicine department because she suffered from low back pain for 2 months. She had no other relevant medical history. Her height and weight were 170 cm, 64 kg (body mass index [BMI]: 22.1). Her pain was dull and pricking, which persisted throughout the day in any posture and was aggravated by the sitting position. The pain affected the lower back, both anterior superior iliac spines, and the lateral side of the right thigh. The patient scored the pain as 8 out of 10 on the numeric rating scale (NRS). The Oswestry disability index (ODI) score was 55, indicating severe disability. The physical examination showed no motor or sensory issues. On lumbar spine MRI, there was no HIZ on the L5-S1 disc area, and mild disc bulging with an annular fissure was discovered at the same level (Fig. 1). The patient had received posterior medial branch blocks (PMBB) 3 times and percutaneous epidural adhesiolysis at another hospital; however, she was never administered PRF. The PMBB effect only lasted for 6 to 10 hours. Therefore, she was referred to our hospital for posterior medial branch CRF. We performed PMBB at the L3-5 levels to confirm that this method was ineffective. Through the medical history, physical examination, and MRI, we determined that the pain originated from the L5-S1 IDD.

### 2.2 Procedure technique

**Figure 2:**
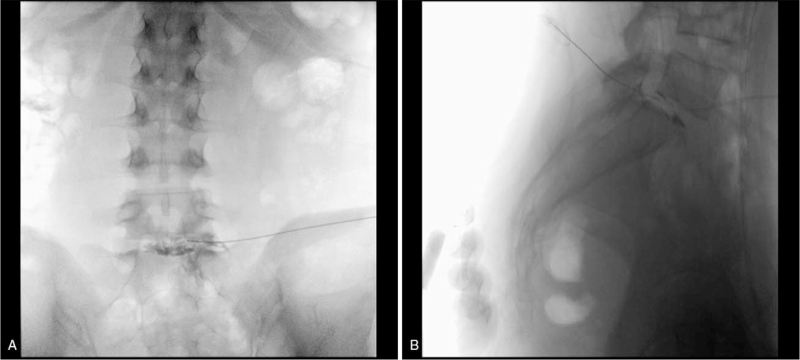
A pulsed radiofrequency straight-tip cannula was inserted into the external one-third L5-S1 annulus; the correct positioning was confirmed with a contrast agent under C-arm fluoroscopy.

Two months after the symptom onset, we performed intradiscal PRF on the L5-S1 disc under C-arm fluoroscopy guidance (Ziehm Vision, Nuremberg, Germany). We used a 20-gauge sharp straight-tip cannula (RF Insulated Hybrid Cannula, 150 mm with a 20-mm active tip; DIROS Technology Inc., Markham, ON, Canada). The patient was in a prone position with back flexion for the surgery. After confirming the target disc under C-arm fluoroscopy, the skin entry point was set 12 cm laterally from the spinous process, and the needle penetrated the L5 superior articular process at an angle. The cannula tip was placed in the dorsal area of the target disc. It was confirmed that the pain was elicited with 50 Hz 0.5 V stimulation, and there was no response to the motor at 2 Hz 2 V. Then, the cannula was carefully introduced into the L5-S1 disc. From the anteroposterior and lateral fluoroscopic view, we determined that the cannula tip was correctly placed, and PRF was performed with an RF generator (Cosman G4, Burlington, MA, USA) (Fig. 2). The PRF parameters were 5 Hz, 20-ms pulse width, and 70 V for 15 minutes. While performing this therapy, we carefully monitored the electrode tip temperature to avoid exceeding 42°C. The impedance of the cannula decreased during PRF from 200 to 160 Ω.

### 2.3 Outcome

Immediately after treatment, her pain subsided (NRS 0), and at the 1-month follow-up visit, the patient reported that her discogenic back pain was completely relieved (NRS 0). The ODI significantly improved to 14 points. She also reported that she could sit for a long time because of pain reduction. No adverse effects were noted from intradiscal PRF.

## 3 Discussion

We reported a case of IDD that responded to intradiscal PRF. This patient's pain score improved from 8 to 0, and the ODI improved from 55 to 14 with the procedure; the improvement was maintained after 3 months of follow-up.

The intervertebral disc is innervated by the sinuvertebral nerve, a branch of the gray ramus communicans. Most of the sinuvertebral nerves are located at the periphery of the annulus fibrosus; however, in discogenic pain, these nerves penetrate the annulus fibrosus, exacerbating the pain.^[13]^ Interventions for IDD include intradiscal steroid instillation, radiofrequency denervation, intradiscal electrothermal therapy (IDET), and surgical management.^[8,14–17]^ IDET contracts the disc at high temperature; in contrast, PRF maintains the temperature below 42°C, avoiding structural degeneration.

The exact mechanism of action of PRF on IDD is still unclear; however, 2 mechanisms were proposed.^[18–21]^ The first is that a strong electric field is created when high-voltage PRF is delivered into the disc, with a biological effect on nerve endings. PRF decreases the activity of microglial cells in the dorsal horn; as microglial cells regulate pain signals through several cytokines and chemokines, modulating these cells may control chronic pain. The second possible mechanism is that PRF stimulates various descending inhibitory pathways, including serotonergic and noradrenergic pathways.

Although provocative discography is typically required to diagnose discogenic pain, recent studies have suggested that the discal pain origin can be suspected based on clinical symptoms and MRI findings; thus, provocative discography could be avoided.^[3,22,23]^ In addition, previous studies showing that PRF is effective on discogenic pain were performed after a provocative discography diagnosis or after 6 months of ineffective conservative treatment. In the present case, the diagnosis was obtained through history-taking, physical examination, and MRI findings, and PRF was performed. As a result, there was an improvement in daily life functions measured by NRS and ODI at 3 months after intradiscal PRF, though additional studies are needed to confirm the long-term outcome. In addition, the radiofrequency energy duration was set to 15 minutes in this case; however, according to recent studies, the effect varies based on the treatment duration,^[24,25]^ and additional research is needed.

In conclusion, intradiscal PRF appears an effective treatment modality for IDD patients diagnosed through clinical history, physical examination, and MRI findings, even without an HIZ.

## Acknowledgment

We would like to thank Editage (www.editage.co.kr) for English language editing.

## Author contributions

**Conceptualization:** Jin Yong Jung.

**Data curation:** Dong Hyuck Kim, Kyung Wook Jeong, Jin Yong Jung.

**Formal analysis:** Dong Hyuck Kim, Kyung Wook Jeong.

**Investigation:** Kyung Wook Jeong, Wonyeong Jo, So Young Lee, Jin Yong Jung.

**Methodology:** Dong Hyuck Kim, Kyung Wook Jeong, Wonyeong Jo, Jin Yong Jung.

**Resources:** Jin Yong Jung.

**Supervision:** Jung A Im, Jin Yong Jung.

**Validation:** Wonyeong Jo, So Young Lee, Jin Yong Jung.

**Visualization:** Wonyeong Jo.

**Writing – original draft:** Dong Hyuck Kim, Kyung Wook Jeong.

**Writing – review & editing:** Jin Yong Jung.
